# STRATEGIES AND GOALS PREDICT DAILY EMOTION REGULATION EFFICACY BUT REAPPRAISAL USE IS LESS PREDICTIVE ACROSS AGE

**DOI:** 10.1093/geroni/igae098.2708

**Published:** 2024-12-31

**Authors:** Bruna Martins-Klein, Ziyuan Chen, Irina Orlovsky, Elinor Waite, Katherine Dixon-Gordon

**Affiliations:** University of Southern California, Los Angeles, California, United States; University of Southern California, Los Angeles, California, United States; University of Massachusetts Amherst, Amherst, Massachusetts, United States; University of Massachusetts Amherst, Amherst, Massachusetts, United States; University of Massachusetts Amherst, Amherst, Massachusetts, United States

## Abstract

Socioemotional goals shift with age, as older adults prioritize regulating emotion to maximize positive affect (hedonic goals) more so than to complete tasks (instrumental goals). Hedonic goals are associated with greater reappraisal use (aka positive reinterpretation), and instrumental goals predict suppression use in daily life, but age differences in the impact of strategies and goals on subjective daily emotion regulation effectiveness has not been previously explored. In this study, 113 participants aged 20-72 years old (M = 38.2; SD = 11.4) completed a 14-day MTurk daily diary on emotion regulation goals, reappraisal and suppression use, and subjective emotion regulation effectiveness in response to daily stressors. Multilevel models nested within day revealed positive effects of age (β=0.03, p<.05) and reappraisal use (β=0.11), and a negative effect of suppression use on subjective regulation effectiveness (β=-0.07, ps<.001). Interestingly, hedonic and instrumental goals positively predicted effectiveness even when controlling for strategy and age effects (ps<.001). In addition, a significant age x reappraisal interaction revealed positive associations of reappraisal use on effectiveness that were stronger for younger adults (β=0.18) than for middle aged adults (β=0.13, ps<.001), and not significant for older adults (p=.47). Thus, goals and strategies uniquely influence emotion regulation in daily life, but reappraisal use is unrelated to late-life regulatory efficacy. Future work should investigate whether increasing salience and awareness of emotion regulation goals may be a key interventional target, which could have important clinical implications for promoting emotion regulation efficacy in older adulthood.

